# Cap-preserving SMILE Enhancement Surgery

**DOI:** 10.1186/s12886-018-0712-7

**Published:** 2018-02-17

**Authors:** Ahmed N. Sedky, Sherine S. Wahba, Maged M. Roshdy, Nermeen R. Ayaad

**Affiliations:** 1 Eye Subspecialty Center, Cairo, Egypt, 18 Elkhalifa Elmamoun Street, Heliopolis, Cairo, Egypt; 2 Ain Shams University, Al Watany Eye Hospital and Watany Research and Development Center (WRDC), Cairo, Egypt; 3 Eye Subspecialty Center, Cairo, Egypt

**Keywords:** SMILE, SMILE enhancement, Cap-preserving SMILE enhancement, Re-SMILE

## Abstract

**Background:**

Different enhancement procedures have been suggested for reduction of residual refractive errors after SMILE. The aim of this study is to evaluate an improved cap-preserving technique for enhancement after SMILE (Re-SMILE).

**Methods:**

A retrospective case series was conducted at Eye subspecialty center, Cairo, Egypt on 9 eyes with myopia or myopic astigmatism (spherical equivalent – 8.0 and − 12.0D). undergoing SMILE procedure and needed second interference. This was either because the more myopic meridian was more than − 10.0 D and therefore planned to have two-steps procedure (six eyes) or because of under correction needing enhancement (three eyes). Assessment after the primary SMILE procedure was conducted at 1 day, 1 week, 1 month and 3 months postoperatively. Assessment after Re-SMILE was conducted at 1 day, 1 week, 1 month, 3 months, 6 months and 1 year postoperatively. The assessments included full ophthalmic examination, objective and subjective refraction, and rotating Scheimpflug camera imaging.

**Results:**

Preoperatively, the mean refractive spherical equivalent (MRSE) values were: − 9.36 ± 0. 89. After primary SMILE it was − 2.18 ± 0.71. After Re-SMILE it was − 0.13 ± 0.68. MRSE was significantly improved after both procedures (*P* < 0.01). The safety index of primary SMILE cases was 1.65 ± 0.62 and for Re-SMILE 1.13 ± 0.34 and the efficacy index was 1.14 ± 0.24 after primary SMILE and 1.11 ± 0.26 after Re-SMILE.

**Conclusion:**

Centered cap-preserving Re-SMILE is an effective procedure in reducing residual refractive errors after primary SMILE in high myopes.

## Background

Laser in-situ keratomileusis (LASIK) and photorefractive keratectomy (PRK) have been the two standard keratorefractive procedures. Small incision lenticule extraction (SMILE) was developed to reduce the corneal biomechanical compromise of LASIK and PRK. In numerous studies, the SMILE procedure was shown to be safe, predictable, and effective in treating myopia and myopic astigmatism [1–3].

As in any refractive procedure, residual refractive errors might occur. For example, Hjortdal et al. published that 20% of eyes have ≥0.5 D and 6% have ≥1.0 D of residual refractive error three months after SMILE in eyes with moderate to high myopia (mean refractive spherical equivalent (MRSE) -7.19 ± 1.30 D) [4].

Different enhancement procedures have been suggested for the reduction of residual refractive errors after SMILE.

Surface ablation, such as in PRK, causes postoperative pain and can lead to corneal haze. The Circle option, which converts the SMILE cap into a complete LASIK flap followed by excimer laser ablation similar to LASIK, has also been suggested as an enhancement procedure [5]. Another suggestion is the creation of a LASIK flap within the SMILE cap followed by ablation. However, this procedure comes with a risk of crossing the existing cap interface or creation of gas breakthrough [6].

One great benefit of SMILE is the preservation of the anterior layer of the corneal stroma and Bowman’s membrane. All enhancement procedures mentioned above share the disadvantage of losing this SMILE benefit.

Recently, Donate and Thäeron published the first case report on creating a new SMILE lenticule underneath the interface of the primary SMILE procedure with the Sub-Cap-Lenticule-Extraction technique [7]. This method aims to keep the benefits associated with SMILE (i.e. preserving the anterior corneal stroma, including Bowman’s membrane) and re-use the interface of the primary SMILE procedure for the Re-SMILE enhancement procedure.

Through this procedure, it is crucial to achieve a precise geometrical match between the interface of the primary SMILE procedure and the new cuts introduced by Re-SMILE to avoid difficulties associated with lenticule dissection and further subsequent complications.

In our study, our goal was to develop a protocol that provides precise centration of the Re-SMILE procedure with respect to the interface after primary SMILE. The change in the term used in the previous study to the new term “cap-preserving SMILE enhancement surgery” is intended to reflect that the main benefits of the SMILE procedure are preserved.

## Methods

A case series of consecutive 9 eyes of 7 patients was conducted in the Eye Subspecialty Center, Cairo, Egypt. The study adhered to the Tenets of the Declaration of Helsinki. Inclusion criteria were patients asking for laser vision correction with myopia or myopic astigmatism between – 8.0 and − 12.0 diopters (D) undergoing SMILE procedure and needed second interference. This was either because the more myopic meridian was more than − 10.0 D (since this is the maximum treatment allowed by our software version of the VisuMax femtosecond laser (Carl Zeiss Meditec AG, Jena, Germany) and therefore planned to have two-steps procedure (six eyes of four patients) or because of undercorrection needing enhancement (three eyes of three patients). To preserve the SMILE benefits, as less dryness induction, we went for the cap-preserving technique. Exclusion criteria were keratoconus, keratoconus suspects, insufficient corneal thickness to leave 250 μm residual stromal bed, corneal scars, and previous anterior segment surgeries. These patients were not specifically enrolled to receive this surgery for the research aim but when we reached good parameters for a centered cap-preserving Re-SMILE technique we collect and analysed the available data retrospectively.

### Primary SMILE

Preoperative assessment included full ophthalmic examination, objective and subjective refraction including uncorrected distant visual acuity (UDVA) and corrected distant visual acuity (CDVA) and rotating Scheimpflug camera (Pentacam, OCULUS Optikgeräte GmbH, Wetzlar, Germany) imaging.

The primary SMILE surgery was performed using the VisuMax femtosecond laser system with the following parameters used: cap thickness of 100 to 120 μm, cap diameter of 7.5 to 7.7 mm, cap side cut angle 70°, 3 mm incision positioned at 100° and angled at 45°. The lenticule diameter (optical zone) was 6.5 mm, transition zone of 0 to 0.1, and clearance of 0.5 mm, lenticule side cut angle of 90° and edge lenticule thickness of 10 μm. Table 1 shows the other surgical parameters used in the primary SMILE procedure that varied from case to case.

**Table 1:** Showing the surgical parameters used in every patient during the SMILE Procedure

Eye	Initial central corneal thickness	Cap thickness	Planned spherical correction	Planned cylindrical correction	Lenticule central thickness	Calculated residual stromal bed
1	549	100	−8.75	− 1.25	158	291
2	554	100	−9	−1	159	295
3	552	120	−9	−1	172	270
4	533	100	−9	−0.5	139	294
5	577	100	−9	−1	160	317
6	555	120	−10	0	133	302
7	574	120	−10	0	125	329
8	534	100	−10	0	151	283
9	505	100	−6	−2.25	130	275

At the end of the procedure, we performed good massage to the cap, evenly from the center to the periphery, to avoid any potential complications from the mismatch between the bed and the cap like mud-crack type microfolds.

**Fig. 1:**
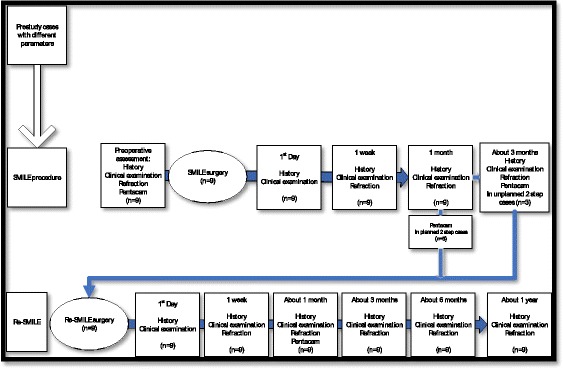
Details of workflow and follow up visits

Postoperative treatment included topical steroids and antibiotics 4 times per day for 10 days and tear substitutes 4 times daily for one to two months. Follow-up visits were on the first day, one week, one month and 3 months postoperatively. Follow-up visits included full ophthalmic examination, objective and subjective refraction, and rotating Scheimpflug camera imaging. In the planned two-step procedures, Pentacam was done after one month. If the refraction was consistent with the predicted one and stable since the first postoperative week the decision was to proceed to RE-SMILE after patient counseling. Figure 1 shows the details of each visit.

### Re-SMILE

The eyes eligible for enhancement were those with expected mean K readings after ablation of not less than 33 D, residual stromal bed of at least 250 μm, and those with no suspicion of ectasia based on tomography.

**Fig. 2:**
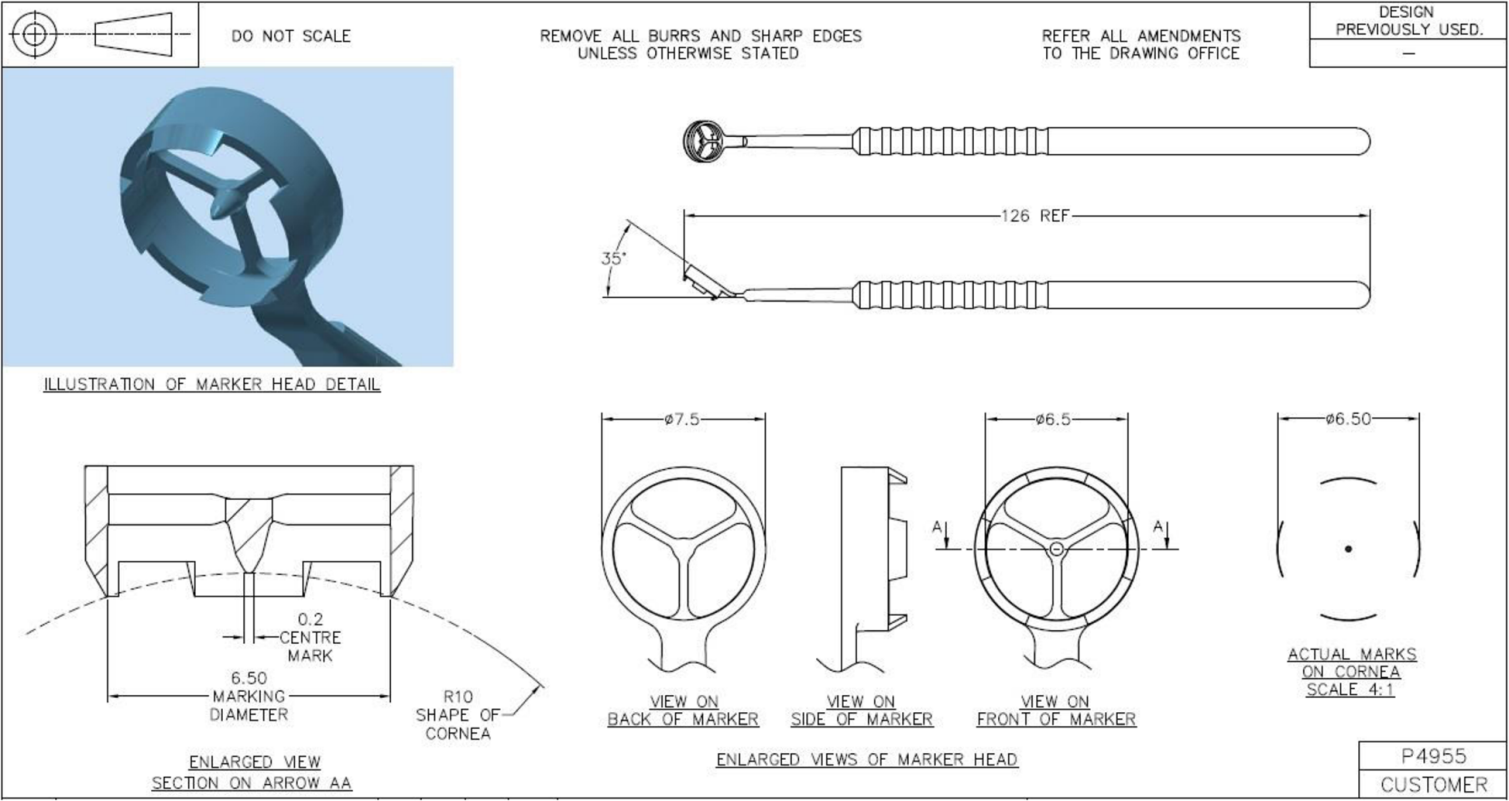
Head of Sedky SMILE Retreatment Centering Marker, 6.5 mm, Duckworth & Kent, Hertfordshire, UK

All Re-SMILE procedures were performed using the same laser device as in the primary SMILE procedure. The Sedky SMILE Retreatment Centering Marker (Fig. 2) was utilized in the centration of the Re-SMILE procedure. Some refractive laser settings were modified with respect to the primary SMILE treatment.

The cap thickness was set to the value used in the primary SMILE treatment. Also, the cap thickness defines the depth of the anterior edge of the lenticule side cut. The parameter for the minimum edge thickness of the lenticule was set at greater or equal to 18 μm. Table 2 shows the variable surgical parameters used in the Re- SMILE procedure.

**Table 2:** Showing the variable surgical parameters used in every patient during the Re-SMILE Procedure

Eye	Central corneal thickness before Re-SMILE	Planned spherical correction	Planned cylindrical correction	Lenticule central thickness	Calculated Residual stromal bed
1	411	−2.25	−0.5	55	256
2	414	0	−2.5	51	263
3	410	−3	0	37	253
4	433	−1.25	−0.5	50	283
5	398	−2	0	30	260
6	427	−2	−1^a^	45	257
7	449	−1.5	−1.5	60	269
8	405	−3.25	0	55	250
9	378	−1	− 0.75	25	250

^a^ *In the eye number 6, although the refractive cylinder was found to be − 2.75 D, only − 1 D was corrected to respect the 250 μm limit*

The programmed sum of optical zone and transitional zone diameters for the Re-SMILE procedure was found to be optimal if it is 0.2 mm less than that of the primary SMILE procedure.

Before arriving at this protocol, a Re-SMILE treatment with a lenticule diameter larger than the primary SMILE lenticule diameter was tested. This setting showed difficult dissection and postoperative intrastromal scaring due to lenticule edge overlapping. The lenticule with the same size as the primary SMILE lenticule demonstrated better postoperative outcome and less intrastromal scaring. However, difficult dissection remained, which might have been due to some mismatch between the Re-SMILE procedure and the primary SMILE.

A new centration marker (Sedky SMILE Retreatment Centering Marker, Duckworth & Kent, Hertfordshire, UK) was developed to improve the centration of the secondary treatment with respect to the primary SMILE treatment (Fig. 2).

The Sedky SMILE Retreatment Centering Marker as shown in Fig. 2 was designed to have four peripheral footplates and central marker pin. The 4 footplates are used to mark the primary cap edge and the central marker pin is used as a docking reference point for the retreatment procedure. The distal 1/3 of the marker handle is designed to have a 35-degree inclination to facilitate usage with the slit lamp at the outpatient clinic or with the built-in VisuMax slit lamp. Both footplates and central marker pin should be inked with a surgical ink (e.g. Viscot Surgical Skin Marker #1404, Viscot Medical, Hanover, NJ, USA) before using the marker. The marker is available in 2 sizes: 6.5 mm for the usage of a 6.7 mm primary lenticule diameter, and 6.3 mm for the usage of a 6.5 mm primary lenticule diameter.

Right before commencing the Re-SMILE procedure, the centration marker was used to mark the patient’s cornea according to the centration of the primary SMILE. During the subsequent docking procedure, these marks were used to achieve precise positioning of the Re-SMILE treatment.

During the actual laser procedure, the surgeon aborted the automated cutting sequence immediately after the laser finished the lenticule side cut.

Then, the new lenticule was removed manually through the primary corneal incision after dissecting the inferior plane. The superior plane of the primary SMILE procedure served as a cap cut for Re-SMILE, which worked because the corneal stroma didn’t heal.

Postoperative treatment after Re-SMILE included topical steroids and antibiotics 4 times per day for 10 days and tear substitutes 4 times per day for one month to two months. Follow-up visits were conducted on the first day, one week, one month, 3 months, 6 months and one year. The assessment included a full ophthalmic examination, objective and subjective refraction and Pentacam imaging. Figure 1 shows the details of each visit.

For each treatment case, the safety index was calculated in decimal units, as postoperative CDVA /preoperative CDVA and the efficacy index as postoperative UDVA /preoperative CDVA [8].

Data were collected, verified, and differences were calculated using Microsoft Excel 2013 (Redmond, Washington, USA). Statistical analyses were performed using MedCalc Statistics (v14.8.1; MedCalc, Belgium) and IBM SPSS Statistics (version 23, SPSS Inc., Chicago, IL, USA). The following statistical tests were performed: calculation of the mean, standard deviation (SD), paired t-test or its non-parametric equivalent Wilcoxon test according to the results of the one-sample Kolmogorov-Smirnov test with Lilliefors significance correction.

## Results

The mean patient age was 28.2 ± 6.3 years (range: 19 to 42). Four eyes were of male patients and the others of female patients.

The MRSE significantly improved after primary SMILE and after Re-SMILE. The refractive cylinder change did not show the same statistical significance, neither after primary SMILE nor after the Re-SMILE procedure as shown in Table 3.

**Table 3:** MRSE, refractive cylinder, UDVA, and CDVA preoperatively, after the initial SMILE and cap-preserving RE-SMILE procedure. t = t-test statistic, p = *p*-value, z = Wilcoxon signed rank statistic

	MRSE(D)	Cylinder(D)	UDVA(logMAR)	CDVA(logMAR)
	Mean ± SD	Mean ± SD	Mean ± SD	Mean ± SD
Preoperative	−9.36 ± 0. 89	−0.78 ± 0.74	0.93 ± 0.20	0.45 ± 0.12
Post primary SMILE	−2.18 ± 0.71	−0.75 ± 0.83	0.40 ± 0.15	0.26 ± 0.20
Significance of changes due to primary SMILE	z = − 2.67 *p* = 0.008	t = − 0.073 *p* = 0.943	z = −2.692 *p* = 0.007	t = 3.875 *p* = 0.005
Post Re-SMILE	−0.13 ± 0.68	−0.53 ± 0.34	0.22 ± 0.12	0.21 ± 0.11
Significance of changes due to Re- SMILE	t = −5.447 *p* = 0.001	t = − 730 *p* = 0.486	t = 3.568 *p* = 0.007	z = −1.187 *p* = 0.235

The UDVA was significantly improved after the primary SMILE and the Re-SMILE procedures. The CDVA shows statistically significant improvement after the primary SMILE but not after Re-SMILE.

**Fig. 3:**
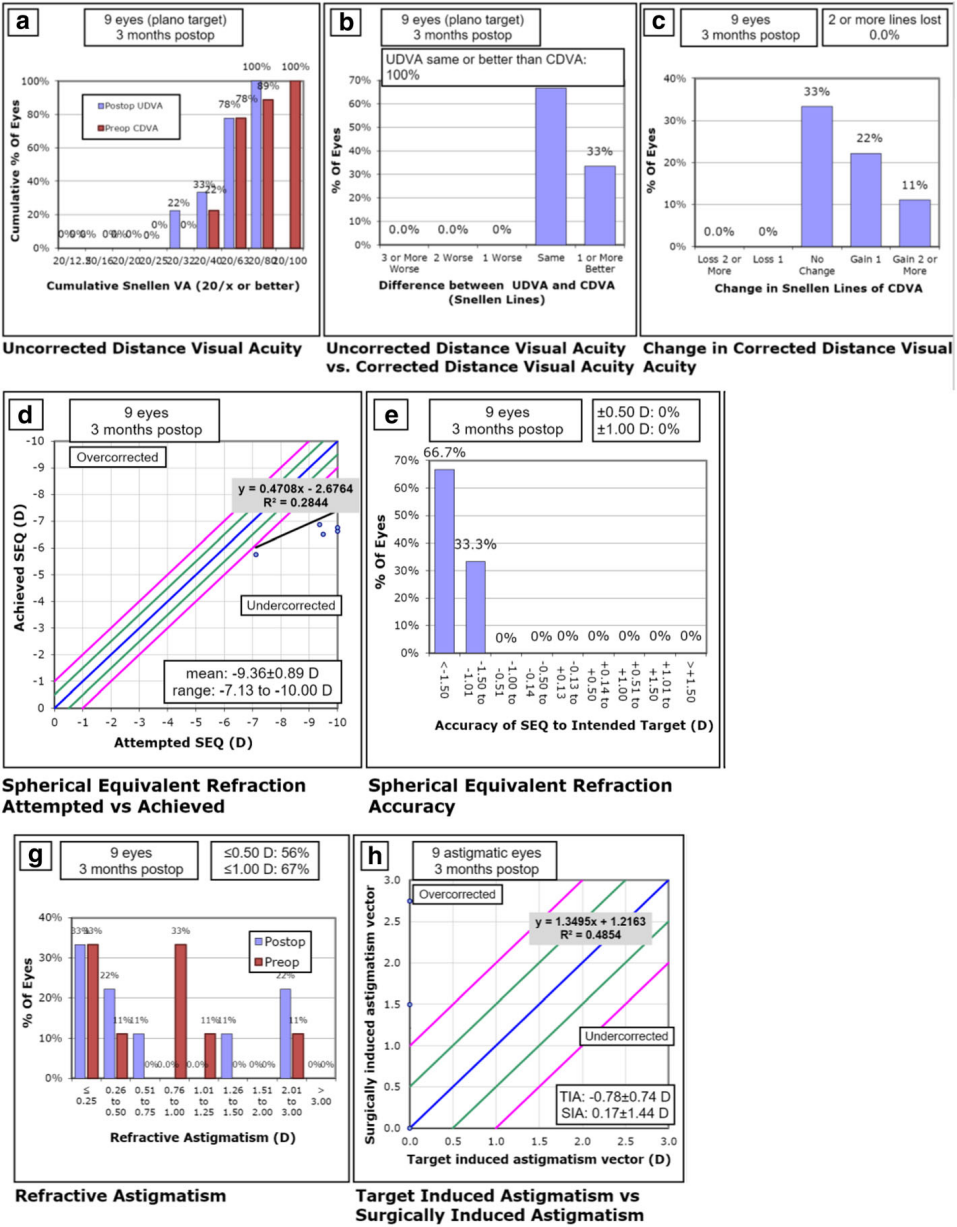
Primary SMILE standardized graphs. **a** Postoperative cumulative uncorrected distance Snellen visual acuity (UDVA) versus preoperative cumulative corrected distance Snellen VISUAL acuity (CDVA). **b** Efficacy of the surgery by comparing postoperative UDVA to preoperative CDVA. **c** Safety of the procedure by comparing pre- and postoperative CDVA. **d** Accuracy of the surgery by comparing attempted versus achieved refractive spherical equivalent (SEQ) and presenting the regression formula describing the relation between them. **e** Accuracy of the surgery by showing the deviation of achieved SEQ compared to attempted SEQ in steps. **g** The residual refractive astigmatism. **h** Accuracy of the surgery by comparing attempted versus achieved refractive cylinder and presenting the regression formula describing the relation between them

**Fig. 4:**
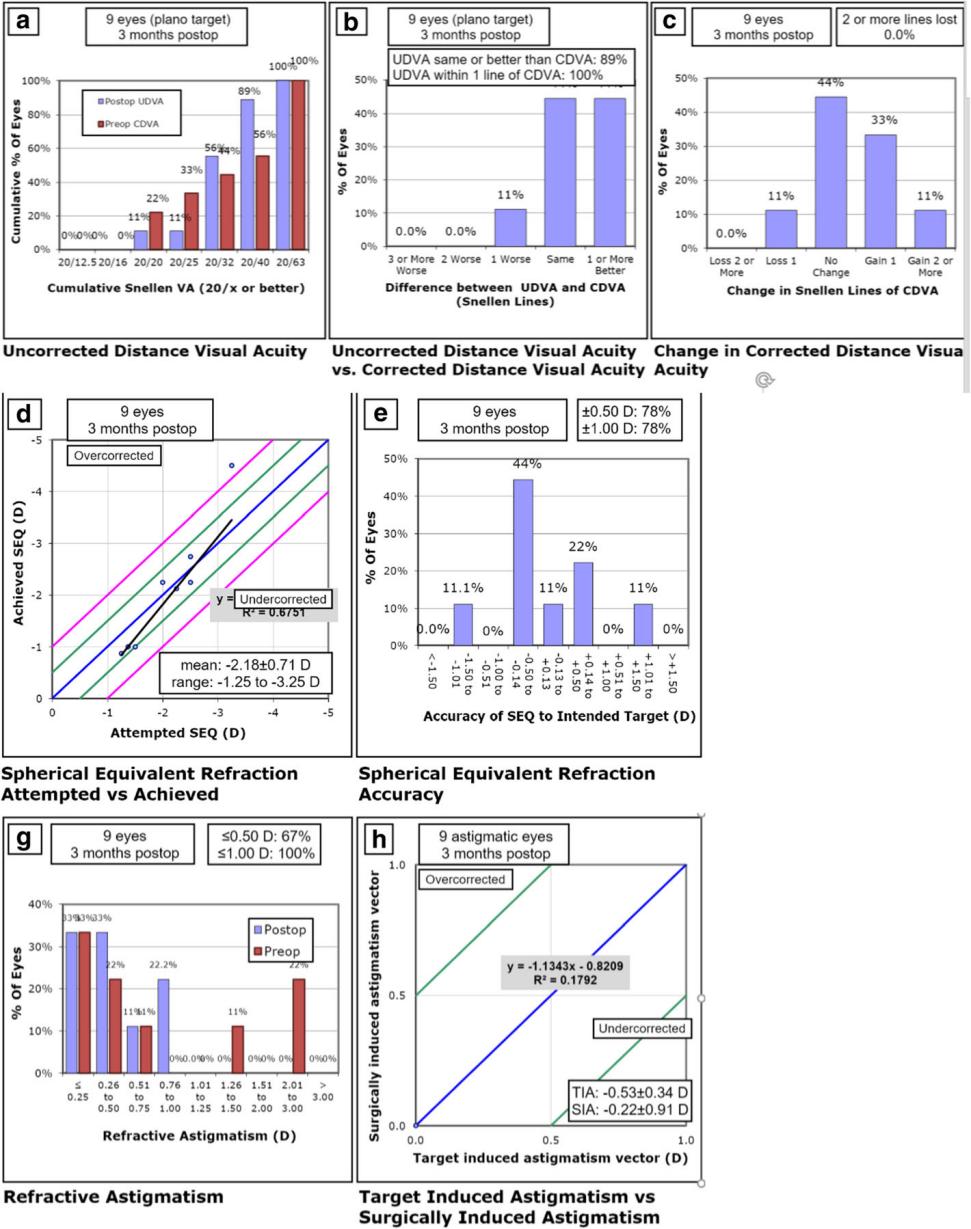
Re-SMILE standardized graphs. **a** Postoperative cumulative uncorrected distance Snellen visual acuity (UDVA) versus preoperative cumulative corrected distance Snellen visual acuity (CDVA). **b** Efficacy of the surgery by comparing postoperative UDVA to preoperative CDVA. **c** Safety of the procedure by comparing pre- and postoperative CDVA. **d** Accuracy of the surgery by comparing attempted versus achieved refractive spherical equivalent (SEQ) and presenting the regression formula describing the relation between them. **e** Accuracy of the surgery by showing the deviation of achived SEQ compared to attempted SEQ in steps. **g** The residual refractive astigmatism. **h** Accuracy of the surgery by comparing attempted versus achieved refractive cylinder and presenting the regression formula describing the relation between them

The standardized graphs of the primary SMILE and of the Re-SMILE procedures presented in Figs. 3 and 4, respectively.

The safety and efficacy indices are summarized in Table 4.

**Table 4:** Safety and efficacy indices in primary SMILE and in Re-SMILE. t = t-test statistic, p = p-value, z = Wilcoxon signed rank statistic

	Mean ± SD	Range	Significance of difference between the two procedures
Primary SMILE Safety Index	1.65 ± 0.62	1.0 to 3.02	t = 1.716, *p* = 0.124
Re-SMILE Safety Index	1.16 ± 0.34	0.67 to 1.67	
Primary SMILE Efficacy Index	1.14 ± 0.24	1.0 to 1.67	z = −0.141, *p* = 0.888
Re-SMILE Efficacy Index	1.11 ± 0.26	0.67 to 1.50	

**Fig. 5:**
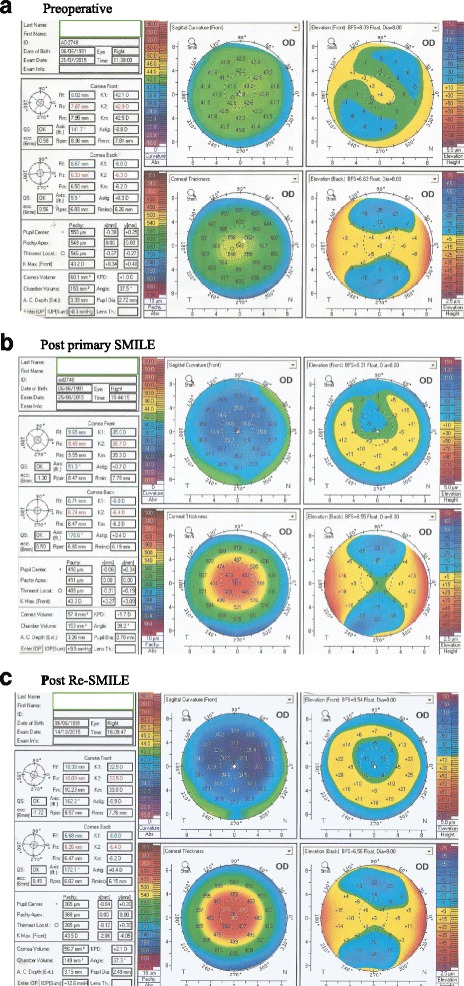
Pentacam scans of one case of planned two-steps procedure: **a** Preoperative **b** Post primary SMILE **c** Post Re-SMILE

The mean Keratometry (k) reading significantly flattened after the primary SMILE procedure (t = 18.725, *P* < 0.001) (Fig. 5). Further reduction was achieved after the Re-SMILE procedure but was not statistically significant (t = 2.245, *P* = 0.055). The mean central corneal thickness (CCT) significantly decreased after the primary SMILE procedure (t = 18.577, P < 0.001). Further reduction was achieved after Re-SMILE but was also not significant (t = 1.494, *P* = 0.173). Detailed results are shown in Table 5.

**Table 5:** K Readings and CCT, before and after the primary SMILE and after the Re-SMILE procedure

	K readings	CCT
	Mean ± SD	Mean ± SD
Preoperative	43.93 ± 1.54	548.11 ± 22.05
Post primary SMILE	37.31 ± 2.27	412.44 ± 21.40
Post Re-SMILE	36.51 ± 2.84	399.44 ± 17.52

No adverse events or side effects occurred during or after primary SMILE and Re-SMILE procedures.

## Discussion

In a recent meta-analysis of SMILE versus femtosecond LASIK composed of 1101 eyes, no significant difference between the two procedures was evident in terms of final MRSE (*P* = 0.72), MRSE within 1 D of the target values (*P* = 0.70), the proportion of eyes losing one or more lines of CDVA after surgery (*P* = 0.69), or those achieving a UDVA of 0.0 logMAR (6/6) or better (*P* = 0.35). However, SMILE was found to be significantly better in terms of tear break-up time (*P* < 0.05) and corneal sensitivity (*P* < 0.003) [8]. Also, SMILE demonstrated a higher predictability compared to femtosecond LASIK. Published reports suggest that with SMILE, 77% to 92% of patients obtain postoperative MRSE within 0.50 D of the intended correction in low to moderate myopic errors. The range decreases slightly for patients with high myopia (77% to 88% within 0.50 D) [1, 4, 9, 10].

A recent review of literature confirmed that SMILE may be of greater benefit in higher refractive corrections [9]. It is reported that between 37% and 98.1% (mean 68.0%) of patients achieved UDVA ≥0.0 logMAR (6/6) and that between 95% and 100% (mean 97.8%) achieved ≥0.3 logMAR (6/12) or better which represents excellent visual outcomes in patients with significant ammetropia [9].

In our study, the time interval between primary SMILE and Re-SMILE was about three months to ensure refraction stability [11].

In our opinion, precise positioning is the key step in the Re-SMILE procedure, because crossing of the new lenticule edge into the edge step in the primary SMILE interface might lead to dissection difficulties and postoperative scarring. Therefore, we developed the Sedky SMILE Retreatment Centering Marker for better centration of the Re-SMILE lenticule with respect to the existing interface of the primary SMILE procedure.

In the study presented, a statistically significant improvement of MRSE after each of the two successive treatment steps occurred. On the other hand, a statistically significant improvement in the refractive cylinder was achieved only by both successive treatments. This may be due to that in the planned two-step surgery cases, full astigmatic correction was not intended in the primary SMILE treatment step, and the residual was reserved for the Re-SMILE treatment. In our case series of two-step treatments, we observed an under correction of cylinder components after both successive treatments. Ivarsen and Hjordal [10] also found a slight under correction of cylinder components in the correction of spherical equivalent and astigmatism in both moderate and high myopia. SMILE patients with high astigmatism (mean cylinder 3.22 ± 0.67 D) were under corrected by 16% of the amplitude of astigmatism leading to the low incidence of patients achieving UDVA of 0.0 logMAR (6/6) or greater. Optimization of personal nomograms and careful orientation control in cases of high cylinder corrections (e.g. by using an orientation marker) may ameliorate this [10].

Our current study shows that after cap-preserving SMILE enhancement surgery with our technique, 89% of enhanced eyes can achieve 0.3 logMAR (6/12) or better UDVA in comparison to only 33% after primary SMILE. Enhanced eyes after cap-preserving SMILE achieved 0.18 logMAR (6/9) or better UDVA in 56% of cases, in comparison to only 11% after primary SMILE. The residual refractive error was − 0.13 ± 0.68 D. All the enhanced eyes had a final MRSE within 1.25 D of intended correction, and 78% were within 0.5 D. All of them became within 1 D of target astigmatism, and 67% were within 0.5 D. At three months postoperatively, results by Chansue et al. were 0.3 logMAR (6/12) or better UDVA in 100% of the eyes, and 0.0 logMAR (6/6) or better in 95.8% of the 24 eyes that were corrected for distance vision [12]. Residual refractive error (spherical equivalent) averaged + 0.1 D. However, their study was conducted to enhance eyes with residual − 0.74 ± 0.80 D of MRSE and − 0.7 ± 0.34 D of refractive cylinder compared to − 2.18 ± 0.71 D of MRSE and − 0.75 ± 0.83 D of refractive cylinder in our study, which resembled more to real life cases suffering clinically significant residual error that deserve second interference.

In our study, the CDVA of some cases improved, especially after primary SMILE correcting the majority of the refractive error. The low preoperative CDVA could be due to most patients’ inexperience with high quality optical correction because of wearing inadequate glasses for a long period of time. In such thick trial set lenses, the minifications and the higher-order aberrations, such as spherical aberration, can compromise the CDVA. Patients often need months after refractive surgery to adapt to the better optical situation resulting in higher visual acuity. It is not uncommon in our practice to find significantly less preoperative CDVA than postoperative CDVA. This is much less encountered in hyperopic patients (magnification effect). However, in some cases, UDVA does not change much. This can be due to induced high-order aberrations by two successive surgeries. Unfortunately, this is not part of our study. Moreover, there is a sort of ceiling effect attained with such high myopic retinae. This also manifested as no improvement with correction post Re-SMILE; the final UDVA is equal to the final CDVA.

To the best of our knowledge, our study presents the first case series results of cap-preserving SMILE enhancements after the first single case report of Donate and Thäeron [7]. We additionally presented here the details of our technique utilizing the Sedky SMILE Retreatment Centering Marker for better centration with determination of both the safety and efficacy index for this case series and the reason for changing the term to cap-preserving SMILE enhancement.

Before reaching the final parameters set for the enhancement lenticule used during this study, we faced some minor complications or difficulties, like when we tried to make the enhancement lenticule same size or bigger than the primary one, dissection of the second lenticule was more difficult particularly over the overlapping edges. Also, during the first cases which are not included in this study, one of the enhancement edge lenticule thickness was thin enough to be torn during dissection. Therefore, we set 18 μm as the minimum enhancement edge lenticule thickness. Creating a new lenticule within the primary lenticule cavity showed no difficulties as long as the new lenticule is well centered especially with the use of Sedky SMILE Retreatment Centering Marker.

A limitation of our study is that we did not evaluate the epithelial thickness before and after the Re-SMILE procedure. However, we accounted for the effect of epithelial thickening by increasing the minimum edge lenticule thickness to at least 18 μm. We also did not evaluate the visual quality. Comparative studies provided mixed results in terms of visual quality. Several authors have found no significant difference between the degree of induced third and fourth order aberrations [13, 14]. Other studies found low induction of HOAs [15]. Both concerns are already under study. More importantly, our technique should be evaluated using a larger sample size to ascertain its safety.

Although the presented technique is not limited to the correction of myopic residual errors in general, it is practically limited to myopic enhancements as long as the commercial indication range of SMILE does not yet allow us to apply the presented approach for hyperopic and mixed astigmatism treatments. Despite the present demand, our new technique increases the need to extend the indication range for SMILE to also include hyperopia and mixed astigmatism.

## Conclusion

SMILE is an effective procedure for the correction of myopia and myopic astigmatism. Enhancement after SMILE can be done using different methods. SMILE enhancement performed using a centered cap-preserving technique (Re-SMILE) is an effective procedure, especially with the use of a special marker to center the cut.

## Abbreviations

CCT: Central corneal thickness
CDVA: Corrected distance visual acuity
D: Diopters
K: Keratometry
LASIK: Laser in-situ keratomileusis
MRSE: Mean refractive spherical equivalent
PRK: Photorefractive keratectomy
SD: Standard deviation
SMILE: Small incision lenticule extraction
UDVA: Uncorrected distance visual acuity
